# Diphtheria in Europe

**DOI:** 10.5588/pha.23.0011

**Published:** 2023-06-21

**Authors:** J. P. Mangion, S. Mancini, C. Bachy, A. de Weggheleire, F. Zamatto

**Affiliations:** Médecins Sans Frontières, Brussels, Belgium

**Keywords:** migration, cutaneous diphtheria, *Corynebacterium diphtheriae*, vaccination

## Abstract

A rising number of diphtheria cases were recorded in Europe in 2022, including in Belgium, within the newly arriving young migrant population. In October 2022, Médecins Sans Frontières (MSF) opened a temporary roadside container-clinic offering free medical consultations. Over 3 months of activity, the temporary clinic detected 147 suspected cases of cutaneous diphtheria with 8 laboratory-confirmed cases growing toxigenic *Corynebacterium diphtheriae*. This was followed by a mobile vaccination campaign, during which 433 individuals living rough in squats and informal shelters were vaccinated. This intervention has shown how even in Europe’s capital, access to preventive and curative medical services remains difficult for those who need it the most. Appropriate access to health services, including routine vaccination, are crucial to improve the health status among migrants.

## Abstract (fr)

Un nombre croissant de cas de diphtérie a été enregistré en Europe en 2022, y compris en Belgique, au sein de la population de jeunes migrants nouvellement arrivés. En octobre 2022, Médecins Sans Frontières (MSF) a ouvert un conteneur-clinique temporaire en bord de route offrant des consultations médicales gratuites. En 3 mois d'activité, la clinique temporaire a détecté 147 cas suspects de diphtérie cutanée et 8 cas confirmés en laboratoire de *Corynebacterium diphtheriae* toxigène. Cette opération a été suivie d'une campagne de vaccination mobile, au cours de laquelle 433 personnes vivant dans la rue, dans des squats et des abris informels, ont été vaccinées. Cette intervention a montré que même dans la capitale de l'Europe, l'accès aux services médicaux préventifs et curatifs reste difficile pour ceux qui en ont le plus besoin. Un accès adéquat aux services de santé, y compris une vaccination de routine, est primordial pour améliorer l'état de santé des migrants.

Between August and December 2022, 24 cases of skin infections caused by toxigenic *Corynebacterium diphtheriae *were reported in Belgium within the young migrant population arriving in the country.^1^ Here, we describe how Médecins Sans Frontières (MSF) responded to this outbreak, adapting the measures to the specificities of a migrant population, initially providing unconditional access to diagnostic and curative interventions, and then, by providing easy access to available vaccines. We share specific recommendations for preventive interventions that might be considered in other countries to pre-empt such outbreaks in the future.

In October 2022, MSF opened a temporary roadside container-clinic offering free medical and psychological consultations in the centre of Brussels in response to the ever-growing number of migrants unable to access shelter and basic medical services. From October to December 2022, more than 2,000 consultations were carried out, with *Sarcoptes scabiei* mite infestation diagnosed in 40% of all patients and upper respiratory tract infections in 11%. In several persons, atypical skin ulcerations were also present, most often on the lower limbs (Figure). Some of these patients described having lesions since passing through migrant camps in the Balkans, while some reported developing lesions when waiting for a shelter in Belgium. Following the confirmation of two cases of *Corynebacterium diphtheriae* skin infections in migrants by the Belgian federal authorities,^2^ local public health authorities collaborated to implement a strategy of presumptively treating all suspected cutaneous diphtheria cases with azithromycin. Additionally, swabs of the ulcers were sent for culture and susceptibility testing to confirm the diagnosis and guide further treatment. Given the intervention setting, isolation of the cases and contact tracing was impossible. During the 3 months of (temporary) clinic activity, 147 suspected cases were treated, with 8 out of 111 wound swabs (7%) growing toxigenic *Corynebacterium diphtheriae*. The average age for the persons who were tested was 25 years (range 14–44), with 84% of persons tested being of Afghan origin. Except for a 31-year-old Syrian, all confirmed cases were from Afghanistan.

**FIGURE:**
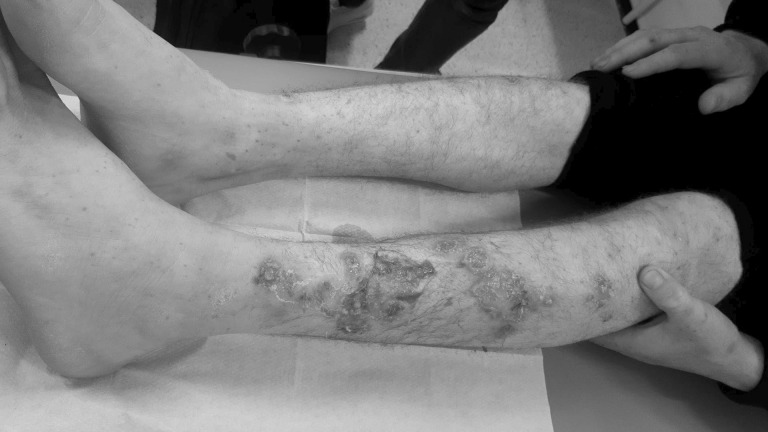
Ulcerative lesions confirmed as cutaneous diphtheria in one of our patients.

In response to the growing numbers of confirmed diphtheria cases and in accordance with the European Centre of Disease Control (Stockholm, Sweden) recommendations,^1^ the Belgian capital’s authorities provided free diphtheria-tetanus-pertussis (Tda-P) and polio vaccines to all newly arrived migrants in a vaccination centre adjacent to the immigration office. However, this fixed vaccination strategy was not able to reach this broader socially disadvantaged group. A 1-week mobile vaccination campaign was therefore carried out by MSF to offer Tda-P and polio vaccination to any migrant, regardless of their legal status living in the larger shelters and informal settlements in Brussels. During this campaign 433 individuals were vaccinated, falling short of the estimated 2,000 migrants living rough in December. The main explanation was not vaccine-hesitancy, but rather individuals not being present at the time the vaccination was offered, as many go only to the squats and in the shelters to sleep at night.

This intervention has shown how even in Europe’s capital, access to preventive and curative medical services remains difficult for those who need it the most. Childhood vaccination coverage for Tda-P in Belgium was at 97.5% for the third dose of the vaccine in 2021,^3^ so the current diphtheria epidemic is not a public health threat. The same is not true for those coming from countries where vaccination coverages have been low for decades. In Afghanistan in the year 2000, when the average patient tested during our consultations was 3 years old, the Tda-P coverage for the third dose was 24%.^4^

The diphtheria cases being seen right now in Europe^1^ highlight the need to rethink the way we look at this old disease that most clinicians have never been confronted with. Consulting contemporary medical textbooks may not provide sufficient insight into the current epidemic, which differs from the classic upper respiratory childhood disease presentation. Instead of the typical respiratory symptoms, most cases are presenting with ulcerative lesions, making it crucial to explore alternative sources of information to better understand and address the current epidemic. These atypical presentations make it harder for clinicians who might not be up to date with the latest epidemiological bulletins to identify cases. In practice, greater effort needs to be made by European authorities to educate clinicians on the challenges of moving populations, as this will remain an important issue in the foreseeable future.

Based on our experience, service provision is not a one-size fits all issue as even when these people present themselves to the local emergency medical services, they are often unable to communicate their needs because of the language and cultural barriers. Ensuring easy access to medical services for all types of migrants remains an essential goal. To achieve this, European states should prioritise providing free and accessible healthcare services to both documented and undocumented migrants while ensuring that seeking healthcare does not result in negative immigration consequences. Factors such as lack of access to healthcare and misinformation, coupled with language barriers can also contribute to low vaccination coverage among migrants. To overcome these barriers, several strategies can be implemented, such as developing tailor-made immunisation services that address the specific needs of the population.^5^ Additionally, outreach and education efforts, community engagement and communication campaigns could be initiated with the support of cultural mediators who work collaboratively with medical staff to enhance the effectiveness of these efforts.

Victims of non-functioning health systems are being welcomed in Europe, only to face other failing health systems that they do not have access to. To prevent future epidemics, the following measures should be implemented: 1) provision of free and unconditional access to health services, including routine vaccination, to all migrants, irrespective of their status; 2) ensuring that accessing health services has no consequences on immigration procedures; 3) the development of specific initiatives to ensure equitable and non-discriminatory access to immunisation programmes, with more work by European policymakers to reach migrants;^6^ 4) training of clinicians in the specificities of migrant medicine in the services targeting migrants; 5) adapting services culturally by providing information in the language that people speak and understand; and 6) making access to cultural mediation a standard in Europe and offering it to patients on a wider scale.
